# The development of a glaucoma-specific health-related quality of life item bank supporting a novel computerized adaptive testing system in Asia

**DOI:** 10.1186/s41687-022-00513-3

**Published:** 2022-10-11

**Authors:** Eva K. Fenwick, Belicia Lim, Ryan E. K. Man, Mani Baskaran, Monisha E. Nongpiur, Chelvin C. A. Sng, Jayant V. Iyer, Rahat Husain, Shamira A. Perera, Tina T. Wong, Jin Rong Low, Olivia Huang Shimin, Katherine Lun, Tin Aung, Ecosse L. Lamoureux

**Affiliations:** 1 Singapore National Eye Centre, Singapore Eye Research Institute (SERI), The Academia, 20 College Road, Level 6, Singapore, 169856 Singapore; 2 Duke–NUS Medical School, National University of Singapore, Singapore, Singapore; 3 National University Health System, Singapore, Singapore; 4 The University of Melbourne, Melbourne, Australia; 5 Medical and Vision Research Foundation, Sankara Nethralaya, Chennai, India

**Keywords:** Glaucoma, Quality of life, Item bank, Computerized adaptive testing, Eyedrops, Qualitative

## Abstract

**Background:**

A glaucoma-specific health-related quality of life (HRQoL) item bank (IB) and computerized adaptive testing (CAT) system relevant to Asian populations is not currently available. We aimed to develop content for an IB focusing on HRQoL domains important to Asian people with glaucoma; and to compare the content coverage of our new instrument with established glaucoma-specific instruments.

**Methods:**

In this qualitative study of glaucoma patients recruited from the Singapore National Eye Centre (November 2018-November 2019), items/domains were generated from: (1) glaucoma-specific questionnaires; (2) published articles; (3) focus groups/semi-structured interviews with glaucoma patients (n = 27); and (4) feedback from glaucoma experts. Data were analyzed using the constant comparative method. Items were systematically refined to a concise set, and pre-tested using cognitive interviews with 27 additional glaucoma patients.

**Results:**

Of the 54 patients (mean ± standard deviation [SD] age 66.9 ± 9.8; 53.7% male), 67 (62.0%), 30 (27.8%), and 11 (10.2%) eyes had primary open angle glaucoma, angle closure glaucoma, and no glaucoma respectively. Eighteen (33.3%), 11 (20.4%), 8 (14.8%), 12 (22.2%), and 5 (9.3%) patients had no, mild, moderate, severe, or advanced/end-stage glaucoma (better eye), respectively. Initially, 311 items within nine HRQoL domains were identified: Visual Symptoms, Ocular Comfort Symptoms, Activity Limitation, Driving, Lighting, Mobility, Psychosocial, Glaucoma management, and Work; however, Driving and Visual Symptoms were subsequently removed during the refinement process. During cognitive interviews, 12, 23 and 10 items were added, dropped and modified, respectively.

**Conclusion:**

Following a rigorous process, we developed a 221-item, 7-domain Asian glaucoma-specific IB. Once operationalised using CAT, this new instrument will enable precise, rapid, and comprehensive assessment of the HRQoL impact of glaucoma and associated treatment efficacy.

**Supplementary Information:**

The online version contains supplementary material available at 10.1186/s41687-022-00513-3.

## Background

The prevalence of glaucoma, a potentially blinding eye condition, is estimated to reach 111.8 million by 2040, with Asia accounting for the largest number of cases worldwide [1]. While primary open angle glaucoma (POAG) is the predominant form of the disease among Caucasians, primary angle-closure glaucoma (PACG), a visually destructive subtype, is a major form of glaucoma in Asians [2]. Independent of visual acuity, glaucoma and its associated treatments can have a negative impact on health-related quality of life (HRQoL) [3–6], which is defined by the International Society for Quality of Life Research (ISOQOL) as the impact of disease and treatment on disability and daily functioning, and the impact of perceived health on an individual's ability to live a fulfilling life. Measurement of HRQoL is traditionally done using patient-reported outcome measures (PROMs), which are now mandated for use in clinical trials by regulatory authorities [7, 8], and are becoming essential to guide clinical care in the era of value-based medicine [9].

While several paper–pencil PROMs are available to measure glaucoma-specific HRQoL [10, 11], a recent systematic review [12] has revealed that most PRO-instruments demonstrated poor developmental quality, particularly a lack of conceptual frameworks and item generation strategies involving the patients' perspective, and psychometric evaluation based largely on classical test theory methods. Moreover, these PROMs usually capture only one or two HRQoL domains and lack content relating to new treatment therapies (e.g. minimally invasive glaucoma surgery [MIGS] and nanotechnology [13]) and other modern trends (e.g. usage of ‘smart’ devices). These issues can be overcome by sophisticated psychometric methods of instrument development, such as item banking and computerized adaptive testing (CAT) [14]. CAT is a ‘smart’ technology that adapts the items (questions) asked based on participants’ responses to previous items [15]. CAT reduces test length without loss of precision by presenting to the respondent targeted items from a calibrated item bank that measures a latent HRQoL construct [15].

A glaucoma-specific HRQoL item bank was recently developed by Matsuura and colleagues [16] in a Japanese population; however, it focused predominantly on activity limitation and a CAT system is not yet available. While our group has already produced an operational CAT for glaucoma (GlauCAT™) [17, 18]. its development was informed by qualitative work with glaucoma patients from Western populations. As such, the content may not be relevant to Asian populations, where glaucoma prevalence and pathophysiology (e.g. POAG vs. PACG) [1, 2], and healthcare systems, treatment regimens, and perceptions of disease burden differ. A time-efficient and focused glaucoma CAT for Asian settings is imperative.

Against this background, our group aimed to develop a glaucoma-specific HRQoL item bank (IB) and CAT system that focuses on the key HRQoL domains most important to Asian patients with glaucoma (GlauCAT™-Asian). This study reports primarily on the generation (Phase 1) and refinement (Phase 2) of domains and items for this new IB. We also compare and contrast the content coverage of our new instrument with other available PROMs that assess the HRQoL impact of glaucoma, with a focus on how GlauCAT™-Asian differs from the GlauCAT™-Western instrument.

## Methods

### Study design and population

English- and Mandarin-speaking patients aged ≥ 40 years with a primary diagnosis of glaucoma (i.e. POAG/PACG) in at least one eye were recruited from the Singapore National Eye Centre (SNEC). Those with other retinal comorbidities including secondary glaucomas, severe cataract, neurological conditions affecting vision, hearing or cognitive impairment (assessed using the 6-CIT questionnaire [19]) were excluded. Patients were purposively recruited to ensure that the spectrum of ethnicity, gender, age, and glaucoma severity was represented.

Phases 1 and 2 of our study were conducted between November 2018 and November 2019 at the SNEC research clinic. The study protocol received approval from the SingHealth Centralised Institutional Review Board (CIRB #2018/2459) and was conducted in accordance with the Declaration of Helsinki. Prior to study participation, written informed consent was obtained from participants by study personnel. Participants were reimbursed SGD $60 and $40 (for focus group and cognitive interviews, respectively) to defray the cost of their participation in this research.

### Assessment of type and severity of glaucoma

Glaucoma subtype, Snellen visual acuity (VA) and visual field (VF) data (both eyes) were extracted from patients’ files. We also conducted binocular Esterman tests using the Humphrey Visual Field Analyzer-3 (Carl Zeiss AG, Jena, Germany). Grading of glaucoma severity was done by glaucoma clinicians and co-authors (MB, MN, and JL) using both the Glaucoma Staging System (GSS) and the Advanced Glaucoma Intervention Study (AGIS) protocol [20, 21] using all available data into better/worse eye with no, mild, moderate, severe, advanced, or end-stage glaucoma. Due to the low number of end-stage glaucoma cases, we combined the advanced/end-stage categories. Snellen VA was converted into equivalent logarithm of the minimum angle of resolution (LogMAR) units and vision impairment (VI) was defined as present if VA ≤ 0.3 LogMAR [22]; and further categorized into mild (> 0.3 LogMAR ≤ 0.48) and moderate/severe (> 0.48 LogMAR).

### Content development for the glaucoma-specific HRQoL IB

#### Phase 1: domain and item generation

We used a ‘‘top-down’’ (theoretical framework informed by our comprehensive literature review and previous experience generating IBs [18]), and ‘‘bottom-up’’ (data-driven) approach for domain and item generation.

##### Literature review

A literature review exploring the impact of HRQoL in patients with glaucoma was performed by authors EF and BL using Pubmed and Google Scholar databases and bibliographies of relevant papers. Keywords included ‘glaucoma’, ‘quality of life’, ‘impact’, ‘functioning’, ‘emotional well-being’, ‘questionnaire’, ‘patient-reported outcome measure’. Findings were used to generate the moderator’s guide for the focus group discussions.

##### Qualitative sessions

Guided by the consolidated criteria for reporting qualitative research (COREQ) guidelines, [23] we conducted a qualitative study in 27 patients across six focus group sessions (four English, two Mandarin). Author EF moderated FGs 1–2 and author BL moderated FGs 3–6. Author BL was note-taker for FGs 1–2, while a Clinical Research Coordinator fluent in both English and Mandarin and trained in qualitative methods was note-taker for FGs 3–6. The composition of each group was arranged to ensure an equal mix of gender, ethnicity and glaucoma severity. Participants answered open-ended questions about how glaucoma had affected different aspects of their HRQoL, i.e. what things were difficult or inconvenient; how this had affected emotional well-being; and the type, frequency and severity of symptoms experienced (see Additional file 1). Participants were also asked which three areas of HRQoL they felt were *most* important in relation to their glaucoma, and these responses were recorded.

Immediately after each focus group (mean = 67 min), the moderator and note-taker debriefed the session and noted if any new themes had emerged. Focus groups were conducted until thematic saturation was reached (i.e. no substantial new themes emerged after two subsequent sessions). Sessions were audio recorded and transcribed verbatim. For sessions conducted in Mandarin, the transcripts were professionally translated to English.

Expert opinion on the impact of glaucoma on patients’ HRQoL was obtained from four glaucoma consultants (7 approached, response rate 57.1%) at SNEC (authors MB, MN, TW and JI). Three open-ended questions were posed and responses collected via email in April 2019.

Patient transcripts and clinician feedback were analysed separately by two researchers (EF and BL) using the constant comparative method [24], and disagreements in coding were adjudicated by a third researcher (EL).

#### Phase 2: item refinement

##### Binning and winnowing

Items generated during Phase 1 were systematically categorized into relevant HRQoL domains based on their content and meaning in a process known as *binning*. In order to reduce the initial large item pools to a more manageable minimally representative set, items were subsequently deleted using a process of *winnowing*, where an expert panel (EF, BL, RM and EL) met face-to-face over two 3-h sessions to remove redundant or duplicate items, and those that did not fit well within the particular HRQoL domain. The panel used a systematic set of criteria developed by the Patient-Reported Outcomes Measurement Information System (PROMIS) group[25] to guide item removal, including:

- Item redundancy—identical wording or too similar in content to another item;
- Item clarity—item confusing, poorly worded, double- or multi-barrelled;
- Item applicability—item too specialised; lacked broad application;
- Item frequency—item did not occur often, or was not well-represented across the four sources of content development.
- Item relevance—precedence was given to items from qualitative patient interviews, as these were considered most likely to accurately reflect patient experiences.

##### Development of item stems, preceding statement and response options

Based on previous instrument development work [26] and empirical evidence [27], items were rated on a 4- or 5-point Likert-type scale. The preceding statement, “Because of your glaucoma or glaucoma treatment…”), timeframe (e.g. “In the past 1 month…”), and item stem to specify the attribute of the QoL construct being measured (e.g. How much difficulty do you have…?”) were also developed, along with short descriptions of each HRQoL domain (see Table 1 for more details on the attribute/timeframe for each HRQoL domain).

**Table 1:** Description of domains and items in our glaucoma-specific quality of life instrument^a^

Domain (n = items)	Definition	Timeframe	Item stem	Response options	Example content
Ocular comfort symptoms (n = 19)	Refers to any sensations or feelings in and around your eyes arising from glaucoma and glaucoma treatment	In the past 1 month…	How much were you bothered by…? How often did you experience…? How often did you feel like…?	*Not at All (4) to A lot (1)* None of the time (4) to All of the time (1)	Pain around eyes, itchy eyes, headache
Activity limitation (n = 72)	Difficulties performing daily activities because of glaucoma or poor vision	None	How much difficulty do you have…?	*None (5)* to *Unable to do because of my vision (1)*	Reading (i.e. from a computer screen, street signs, bus numbers), cooking, finding things dropped on the floor, playing different sports
Lighting (n = 15)	How glaucoma and associated vision problems affect ability to do things in different lighting conditions	None	How much difficulty do you have…?	*None (5)* to *Unable to do because of my vision (1)*	Seeing under fluorescent or indoor lighting, driving in the day/night, going down steps in dim lighting
Mobility (n = 19)	The impact of glaucoma and associated vision loss in moving around the community independently	None	How much difficulty do you have…?	*None (5)* to* Unable to do because of my vision (1)*	Walking (i.e. around unfamiliar areas, on uneven ground), noticing things or people to the left or right, getting on or off public transport
Psychosocial (n = 55)	Describes any concerns about or emotional reactions to having glaucoma and associated vision loss; as well as the impact of glaucoma on social life and personal relationships	In the past 1 month…	How concerned were you about…? How often did you feel…?	*Not at all (5)* to *Extremely (1)* None of the time (5) to All of the time (1)	Falling, your eyesight getting worse, being burden to your family Frustrated, depressed, helpless, socially isolated
Glaucoma management (n = 28)	Difficulties and concerns surrounding glaucoma treatment, such as financial impact, difficulties in constantly administering eyedrops, etc	None	How difficult is…? How concerned are you about…?	*Not at all (5)* to *Extremely (1)*	Consistently administering the right amount of eyedrops, attending frequent appointments, the ongoing costs of glaucoma eyedrops
Work (n = 13)	Work performance and financial impact of glaucoma	Currently…	How much difficulty do you have…? How concerned are you about…?	*None (5)* to *Unable to do because of my vision (1)* Not at all (5) to Extremely (1)	Keeping up at work, having limitations on the types of job

^a^Brief descriptions of key terms were provided as part of initial participant instructions to ensure they were understood consistently by all participants

##### Cognitive interviews

Following the development of the item pools, cognitive interviews were conducted with glaucoma patients, who were recruited from SNEC using the same eligibility criteria as described above. Cognitive interviews allow any issues to be addressed prior to large-scale testing. The “think-aloud” and “verbal-probing” methods [28] were used, which allowed patients’ comprehension of the item stems, items and response options to be tested using open-ended questions (e.g. “I noticed you had to take time to understand the question. Can you tell me why this was?”). As the preliminary instrument was long (n = 232 items), it was not feasible to test the entire set. As such, 30 questions with high potential for response errors were shortlisted by the study team for testing.

Interviews were conducted in rounds of 3–4 participants and feedback was iteratively incorporated after each round. Changes were re-tested in a new batch of participants until no new issues emerged, resulting in a total of 19 glaucoma patients completing the cognitive interviews. Finally, an additional 8 participants completed the full questionnaire (i.e. all 232 items) as a ‘dry-run’ until no new issues emerged, resulting in a final sample of 27 patients.

## Results

### Phase 1: domain and item generation

#### Literature review

Based on our literature review, 77 and 44 items within multiple domains of HRQoL were extracted from four relevant qualitative papers [18, 29–31] and four reviews [32–35], and 11 questionnaires or IBs [10, 11, 16, 18, 36–42], respectively (Table 2).

**Table 2:** Items generated across four sources of content development (Phase 1)

Source of content development	Number of novel items generated
Qualitative (n = 4) & review articles (n = 4)	77
Validated patient reported outcome measures (n = 11)	44
Qualitative sessions with patients (n = 27 patients)	158
Qualitative sessions with experts (n = 4 experts)	32
Total	311

#### Qualitative sessions

Of the 27 patients (mean ± standard deviation [SD] age 67.9 ± 8.2; 48.1% male, 81.5% Chinese), who participated in the focus groups, nine (33.3%), five (18.5%), two (7.4%), eight (29.6%) and three (11.1%) had no, mild, moderate, severe, advanced/end-stage glaucoma in the better eye, respectively (Table 3). Most participants (n = 26, 96.3%) had received topical medication in at least one eye, with nine (33.3%) and 11 (40.7%) receiving laser and surgery (e.g., trabeculectomy, minimally invasive glaucoma surgery, aqueous shunts), respectively.

**Table 3:** Sociodemographic and clinical characteristics of the 27 participants in Phase 1

Variable	N	%
*Gender*		
Male	13	48.1%
*Age (Years)*		
40—49	1	3.7%
50—59	3	11.1%
60—69	12	44.4%
69 <	11	40.7%
*Ethnicity*		
Chinese	22	81.5%
Malay	2	7.4%
Indian	3	11.1%
*Duration of glaucoma (years)*		
0–2	2	27.4%
3–5	10	37.0%
6–10	6	22.2%
11–15	5	18.5%
> 15	4	14.8%
*Allergic reactions/side effects to medication*		
Yes	10	37.0%
No	17	63.0%
*Glaucoma type (per eye)*		
POAG/ NTG	33	61.1%
PACG	18	33.3%
None	3	5.6%
*Glaucoma severity (better eye)*		
None	9	33.3%
Mild	5	18.5%
Moderate	2	7.4%
Severe	8	29.6%
Advanced/End-stage	3	11.1%
*Glaucoma severity (worse eye)*		
Mild	4	14.8%
Moderate	7	25.9%
Severe	10	37.0%
Advanced/End-stage	6	22.2%
*Glaucoma treatments (in at least one eye)*		
Topical medication	26	96.3%
Laser	9	33.3%
Surgery	11	40.7%
*Vision impairment (better eye)*		
None (≤ 0.3 LogMAR or ≤ 20/40 Snellen)	26	96.3%
Mild (> 0.3 LogMAR ≤ 0.48 or > 20/40 Snellen ≤ 20/60)	1	3.7%
Moderate/severe (> 0.48 LogMAR or > 20/60 Snellen)	0	0.0%
*Vision impairment (worse eye)*		
None (≤ 0.3 LogMAR or ≤ 20/40 Snellen)	15	55.6%
Mild (> 0.3 LogMAR ≤ 0.48 or > 20/40 Snellen ≤ 20/60)	2	7.4%
Moderate/severe (> 0.48 LogMAR or > 20/60 Snellen)	10	37.0%
*Marital status*		
Single	3	11.1%
Married	20	74.1%
Divorced/separated/widowed	4	14.8%
*Highest education level*^*a*^		
Primary	5	20.8%
Secondary	11	45.8%
A Level	3	12.5%
Polytechnic/Diploma/ Vocational Training	2	8.3%
University or higher	2	8.3%
*Employment status*		
Working	20	74.1%
Not working	7	25.9%
*Chronic health conditions*^*a*^		
Hypertension	10	37.0%
Dyslipidaemia	12	44.4%
Diabetes	5	18.5%
Heart attack	1	3.73%
Stroke	1	3.7%

Continuous variables	Mean	SD
Age (years)	67.9	8.2
Presenting VA (better eye), LogMAR; Snellen	0.11; 20/25	0.11; 20/25
Presenting VA (worse eye), LogMAR; Snellen	0.37; 20/40	0.21; 20/32
Visual fields (better eye), mean deviation	− 10.36	9.69
Visual fields (worse eye), mean deviation	− 16.83	9.74
No. of topical treatments within past 6 months	1.8	0.9

^a^Percentages for some variables may not equal 100% due to missing data or participants selecting > 1 category

*LogMAR* logarithm of the minimal angle of resolution; *NTG* normal tension glaucoma; *PACG* primary angle closure glaucoma; *POAG* primary open angle glaucoma; *SD* standard deviation; *SGD* Singapore dollars; *VA* visual acuity

Following thematic analysis, we isolated 311 unique items, across nine domains (Table 4, row 1 ‘initial pools’), namely Visual Symptoms; Ocular Comfort Symptoms; Activity Limitation; Driving; Lighting; Mobility; Psychosocial (including concerns, emotional reactions and social well-being); and Glaucoma Management (including challenges and concerns relating to glaucoma treatment and attending appointments); and Work.

**Table 4:** The process of refining the initial item pools to the final pilot instrument (Phase 2)

	VS	OS	AL	DV	LT	MB	PS	GM	WK	Total
Initial pools	18	21	73	18	10	22	108	18	23	311
After domain ranking	18	21	79	–	16	20	69	18	20	261
Binning & winnowing #1	15	19	79	–	16	20	69	16	20	254
Binning & winnowing #2	–	20	77	-	16	20	70	16	13	232
Cognitive interviews	–	19	72	–	15	19	55	28	13	221

*VS* visual symptoms; *OS* ocular comfort symptoms; *AL* activity limitation; *DV* driving; *LT* lighting; *MB* mobility; *PS* psychosocial; *GM* glaucoma management; *WK* work

The three most important HRQoL domains listed by our focus group patients were Ocular Comfort Symptoms, Mobility and Psychosocial, with Activity limitation and Glaucoma Management also frequently mentioned. However, Driving was rarely listed by patients as important, most likely due to the low number of elderly people driving in Singapore; as such, we dissolved the Driving domain (moving some items to other domains like Activity limitation or Lighting). This resulted in 261 items across eight domains (Table 4, row 2 ‘After domain ranking’). Themes for each HRQoL domain are briefly outlined below, with more information and supporting quotes provided in Additional file 2.

The most commonly reported visual symptoms by our glaucoma patients were blurred vision and ‘blocking’ of vision (i.e. a sense of obstruction, vision being cut off). Commonly mentioned ocular comfort symptoms were feeling like there was something in their eyes (i.e. a foreign body sensation) and a sticky sensation around eyelashes or eyelids. Some patients reported that administering eyedrops was tiresome, while others worried whether their treatment plan was effective. Many patients found reading small print (e.g. letters or bills), using internet banking, walking on uneven ground and seeing people or objects coming towards them daunting due to their vision. Several patients reported that dim lighting and/or glare affected their ability to perform daily activities. Difficulties reading and working on a computer screen for long hours impacted the work performance of some patients, which often strained work relationships with colleagues or supervisors. A universal fear reported by glaucoma patients was further loss of vision and eventual blindness. Most patients also expressed safety concerns, like falling, tripping, or bumping into people or objects.

#### Item generation summary

At the conclusion of Phase 1, the number of unique items generated from four separate sources was 311 (Table 4, row 1 ‘initial pools’), comprising 77, 44, 158 and 32 items from eight papers, 11 glaucoma-specific questionnaires, patient focus groups, and expert feedback, respectively.

### Phase 2: item refinement

#### Binning and winnowing

The eight domains were evaluated during two sessions of binning and winnowing, during which, the expert panel decided to remove Visual Symptoms because the items were deemed to function more as a checklist than a latent construct and could be quickly captured during history taking. Certain items from the Visual Symptoms domain (e.g. difficulty telling the difference between similar tones and shades and difficulty with seeing haloes around lights at night) were moved to other domains, namely Activity Limitation and Lighting. All remaining items were reviewed for importance, clarity and relevance after which they were either preserved, redirected to a different domain, or deleted. The number of items was eventually reduced from 261 to 232 (Table 4, rows 3 & 4 ‘Binning & winnowing’).

#### Cognitive interviews

Of the 27 patients (mean ± SD age 65.8 ± 11.3; 59.3% male, 88.9% Chinese) who participated in the cognitive interviews, nine (33.3%), six (22.2%), six (22.2%), four (14.8%), and two (7.4%) had no, mild, moderate, severe, advanced/end-stage glaucoma in the better eye, respectively (see Additional file 3). Twenty-one patients (77.8%) had received topical medication in at least one eye, with seven (25.9%) and eight (29.6%) receiving laser or surgery in at least one eye, respectively.

Based on the feedback from the cognitive interviews and dry-runs, the study team made several amendments (Table 5) including addition (n = 12), deletion (n = 23) and modification of items and response options (n = 10), resulting in a final 7-domain, 221-item item bank (Activity Limitation, n = 72 Lighting, n = 15; Mobility, n = 19; Psychosocial, n = 55; Ocular Comfort Symptoms, n = 19; Glaucoma Management, n = 28; Work, n = 13) (Table 4, row 5 ‘Cognitive interviews’).

**Table 5:** Examples of item modifications following the cognitive interview process

Quality of life domain	Item	Type of change	Reason for change
Ocular comfort symptoms	‘How often have you experienced sunken eyes, or your eyes feeling sunken?’	Re-phrased	Feedback suggested the question was hard to comprehend and the item stem was modified and rephrased for clarity Final item: ‘How much were you bothered by sunken eyes appearance?’
Activity limitation	‘How much difficulty do you have playing indoor sports, e.g. badminton, bowling, gym sessions, table tennis?’	Edited examples	Participants’ suggestions on common types of indoor sports
Activity limitation	‘How much difficulty do you have visually scanning a document for information?’	Re-phrased	After several rounds of testing different wordings and phrasings, the question was modified to ‘How much difficulty do you have finding information in a document’
Lighting	‘How much difficulty do you have getting enough light to see?’	Deleted	Patients found the question confusing
Mobility	‘How much difficulty do you have seeing objects coming towards you, e.g. cars, bikes, scooters?’	Added	Patient suggestion
Psychosocial	‘In the past 4 weeks, how often did you feel like you wanted to give up on your glaucoma treatment?’	Added	A constant theme noticed in patients who have been diagnosed with glaucoma for several years

#### Comparison of GlauCAT™-Asian and other PROMs used to measure HRQoL in glaucoma

**Fig. 1:**
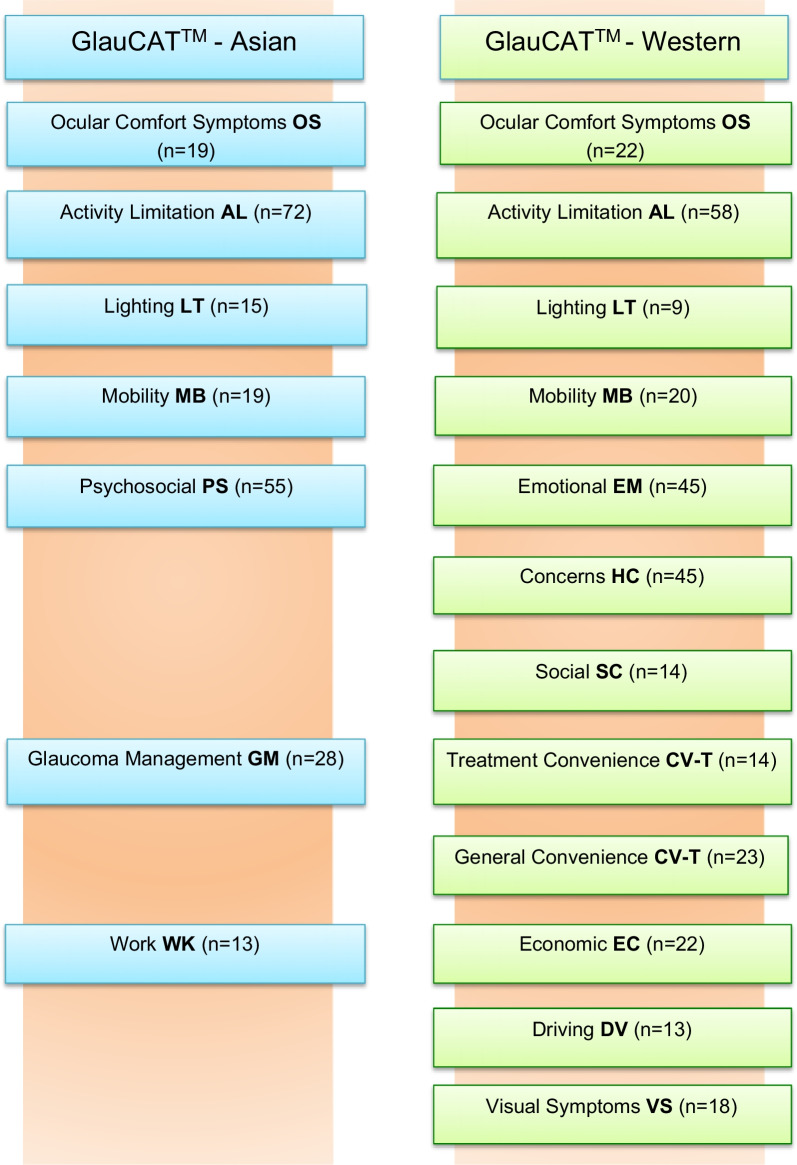
Head-to-head comparison of the domain structure of the new GlauCAT™-Asian and GlauCAT™-Western instruments. This figure shows that four domains—OS, AL, MB and LT—are the same across the two instruments, while the number and content of the remaining domains differs

While Ocular Comfort Symptoms, Activity Limitation, Lighting, and Mobility were present in both the GlauCAT™-Asian and GlauCAT™-Western instruments [17, 18], the remaining domain structure differed (Fig. 1). For example, rather than having three separate domains for Emotional, Concerns and Social as in GlauCAT™-Western, GlauCAT™-Asian consolidated these items under a single ‘Psychosocial’ domain in an effort to streamline the instrument. Similarly, there is no Driving domain in GlauCAT™-Asian, reflecting the fact that few elderly people in Singapore drive.

The domain and item content of nine existing paper–pencil questionnaires used to measure HRQoL in glaucoma [10, 11, 36–42] and the glaucoma IB developed by Matsuura and colleagues[16] was also compared with our new GlauCAT™-Asian instrument (see Additional file 4). Overall, Activity Limitation, Lighting, Mobility, and Psychosocial were reasonably well represented, although the number of items with which to measure the domains in currently available PROMs was limited (median = 8.5, range 3–84). In contrast, Ocular Comfort Symptoms, Glaucoma Management and Work were largely under-represented, with the exception of the Matsuura item bank [16].

## Discussion

Following a robust development and refinement process, we generated 221 items across seven independent glaucoma-specific HRQoL domains. The qualitative sessions with patients were particularly productive for content generation and, while daily activity, mobility and lighting limitations are well-known, issues relating to ocular comfort following treatment (e.g., stickiness around eyelashes), glaucoma management (e.g. concern about having glaucoma surgery) and work (e.g. fear of job loss) are not well captured by paper–pencil glaucoma-specific HRQoL questionnaires. While four domains are common to both GlauCAT™-Asian and GlauCAT™-Western instruments, the remaining domains and item content differs. Once our IBs are calibrated and operationalised via CAT, our new instrument will offer a comprehensive yet efficient measurement of glaucoma-specific HRQoL that is applicable to Asian patients, and will be of relevance to health professionals and researchers with an interest in value-based care.

Our focus group discussions revealed many issues with ocular comfort, especially relating to treatment side effects, such as dry, red and tired eyes, a ‘sunken’ eye appearance, and stickiness and stains around eyelids and lashes, some (but not all) of which have been reported in other studies [43]. While some instruments, such as the Glaucoma Symptom Scale (GSS) [36], and the Comparison of Ophthalmic Medications for Tolerability (COMTOL) scale [41] contain a handful of items relating to glaucoma-specific symptoms, only the GlauCAT™-Western previously developed by our group [17, 18] covers a similar breath of issues. This is important as there may be an association between worse patient-reported side effects and non-adherence to glaucoma medications; however, evidence is equivocal [29, 44–46], which may be due to the lack of an appropriate tool to adequately assess this relationship. A comprehensive and validated glaucoma-specific PROM is hence needed to better assess the treatment side effect-medication adherence relationship.

While the content of the Glaucoma Management domain (GlauCAT™-Asian) and the Convenience-Treatment domain (GlauCAT™-Western) is similar, the Glaucoma Management domain has twice the number of items and covers a broader range of issues. For example, it contains multiple items relating to difficulty administering eyedrops, an issue that has been commonly reported in the literature [29, 47], and one that has been associated with decrements in vision-related HRQoL [48] and non-adherence to medications [46]. This domain also comprises items relating to concerns about having to undergo glaucoma surgery or laser treatment, as well as the financial burden associated with ongoing topical medication use and/or surgery/laser; this is pertinent as inability to afford treatment is a known barrier to adherence [49]. An in-depth and holistic understanding of glaucoma treatment burden from the patient’s perspective using a comprehensive PROM is crucial for clinicians delivering patient-centred care and to improve patient-centred and clinical outcomes.

While work-related issues were reported by some of our focus group participants, this has not been widely reported elsewhere, likely because glaucoma is an age-related condition affecting most people post-retirement. However, with many Singaporeans working well into their 60 s, 25% of our focus group participants (mean age 68 years) were still currently working either part- or full-time. Indeed, work-related issues relating to glaucoma may continue to increase as Singapore plans to raise retirement and re-employment age to 65 and 70, respectively by 2030 [50]. As such, we expect our Work domain to become progressively more relevant in assessing the HRQoL issues that glaucoma patients will invariably face as the workforce ages.

Our finding that glaucoma impacts on daily living activities like reading and getting out and about, especially in challenging lighting conditions [51], is well substantiated in the literature [52], and is reflected by the fact that both GlauCAT™ instruments contain these fundamental HRQoL domains. Another key theme reported in our focus groups, and which mirrors findings from other studies [53], was fear of falling. This important psychological burden has been linked with reduced mobility and physical activity levels and increased fall events [54, 55] in glaucoma patients and suggests that screening for, and developing interventions to minimize fear of falling, may result in important functional improvements for glaucoma patients.

Unlike paper–pencil questionnaires that contain only a handful of items per domain, our HRQoL domains comprise between 13–72 items each and, as such, are able to target the spectrum of patient ‘ability’ level. In the next stage of this multi-phase study, the items will be ranked in terms of relative difficulty in an item bank using Rasch analysis using data from a large patient sample across the spectrum of glaucoma type and severity. Items can then be administered using CAT, which applies an algorithm to administer the best-targeted items from the bank at each stage of the testing process [14], allowing precise estimates of HRQoL to be calculated with relatively few items (depending on the desired measurement precision) [56]. This results in time savings of up to 80% compared to administering equivalent paper–pencil questionnaires [57]. Each glaucoma HRQoL item bank will function independently allowing users to select relevant domains for their sample population (e.g. Glaucoma Management may be most relevant to patients on treatment). However, even if some items are not relevant (e.g., patients not on topical medication cannot answer items about eyedrops), CATs can avoid presenting these items without biasing the overall score. This is a clear advantage over paper–pencil questionnaires, where patients must answer every item regardless of applicability.

In future, the final glaucoma CAT instrument will be able to measure glaucoma-specific HRQoL cross-sectionally, as well as monitor changes over time, for example pre-/post-treatment interventions (surgery, changes in medication regimens) or at routine clinical appointments (e.g., real-world setting). It will be relevant for clinical research studies or trials as a primary or secondary endpoint to measure the magnitude of HRQoL impact in patients across the spectrum of glaucoma, with or without associated VI, and related treatment for glaucoma to support market application. The CATs will be administered on an internet-enabled digital device and will be compliant with accessibility standards for visually impaired patients and the technical requirements of international data security regulatory bodies.

Our substantial qualitative component including 54 patients and four experts is a key strength of our study, as is the psychometric expertise and CAT experience of the development team [56]. We used a systematic and accepted item reduction process [25], and were guided by empirical evidence [27] when generating item stems and response options. Our thorough pre-testing process via cognitive interviewing is also a strength [58]. However, as it was not feasible to conduct in-depth interviews on all 221 items, most were only pre-tested eight times; as such, it is possible that potential issues were missed. Our purposive sampling technique may have introduced selection bias; however, our aim was to obtain detailed information from sub-groups of interest rather from a representative population-based sample. We had fewer Indian and Malay participants compared to Chinese and therefore may have missed culturally-specific content; however, we will raise recruitment numbers for these minority groups in subsequent phases. While our new instrument has been developed in Asian patients, these were limited to one country, Singapore; as such, the content may not be applicable to patients residing in other parts of Asia. Further work to test the cultural appropriateness of GlauCAT™-Asian is required. Finally, we did not gain qualitative feedback from carers who may have provided valuable information on the burden of glaucoma.

## Conclusions

We generated 221 items across seven independent glaucoma-specific HRQoL domains. Once operationalised by CAT, our GlauCAT™-Asian instrument will be useful for clinicians to better understand the impact of glaucoma on patients’ HRQoL, especially once it is fully implemented into routine clinical care and integrated with patients’ electronic medical records; and for researchers to assess the patient-centred impact of novel glaucoma treatment therapies or models of care.

## Supplementary Information

**Additional file 1.** Moderator’s guide for the focus groups. Open-ended questions were asked about how glaucoma, vision loss, and associated glaucoma treatments affected various areas of patients’ health-related quality of life, including activity limitation, symptoms, emotional well-being, social life, and work.

**Additional file 2.** Supporting quotes and excerpts from focus groups showing the diverse and profound impact of glaucoma, related vision loss, and glaucoma treatments on patients’ health-related quality of life.

**Additional file 3.** Sociodemographic and clinical characteristics of the 27 participants who participated in the cognitive interviews to pre-test the GlauCAT™-Asian instrument. Most patients were male, Chinese and had received topical medication in at least one eye.

**Additional file 4.** Comparison of the content (domains and items) covered in the GlauCAT™-Asian instrument and related glaucoma-specific quality of life questionnaires and item banks, showing that some domains (e.g. Activity Limitation, Lighting, Mobility, and Psychosocial) were reasonably well represented in related questionnaires, while others (e.g. Ocular Comfort Symptoms, Glaucoma Management and Work) were less well covered.

## Data Availability

Data reported in this study are available upon reasonable request.
