# Supplementary material for: The development of a glaucoma-specific health-related quality of life item bank supporting a novel computerized adaptive testing system in Asia
Source: J Patient Rep Outcomes. 2022 Oct 11;6:107. doi: 10.1186/s41687-022-00513-3 (PMC9554106; doi:10.1186/s41687-022-00513-3)
Supplement: Supplementary file 1 — Additional file 1. Moderator’s guide for the focus groups. Open-ended questions were asked about how glaucoma, vision loss, and associated glaucoma treatments affected various areas of patients’ health-related quality of life, including activity limitation, symptoms, emotional well-being, social life, and work. [file 41687_2022_513_MOESM1_ESM.docx]

**Additional File 1 – Moderator’s guide for the focus group and interview discussions**

**Introduction and Purpose**

Many thanks for taking the time to join this focus group. My name is [name of moderator]. I am a research fellow/associate/coordinator at the Singapore Eye Research Institute and, today, I will be the ‘moderator’ for this group discussion which means I’ll be running the group and get the conversation going. This is my colleague [name of note-taker] and he/she will be the ‘note-taker’ for this session.

You were chosen to attend this focus group because you have glaucoma. This study aims to understand how your glaucoma, glaucoma treatments and assocaited vision problems impact on all areas of your quality of life. This important information helps doctors, researchers and policy planners to better allocate resources for people with glaucoma.

In this group discussion, we would like to hear your views, experiences and opinions on how your glaucoma affects your quality of life.

**Warm up questions**

*Ground rules:*
*1. Please ensure that your mobile phones are switched to silent mode. We would like the discussion to flow smoothly without any interruptions*

*2. I would appreciate if one person talks at a time, so that everyone will be able to hear what each person has to say.*

*3. I may call on you if I haven’t heard from you in a while. Everyone’s opinions and suggestions are important to my team.*

*4. There are no right or wrong answers. I hope that everyone can respect each other’s opinions and suggestions*

*5. Due to time constrains, I hope that we can stay on topic. If we do digress, I may have to interrupt so that we can cover all the questions in the discussion.*

Let’s start by getting to know each other. Let’s go around the group. Briefly, please let us know your name and how long you have had glaucoma.

**Questions and prompts**

We are interested today in your glaucoma eye problems and how they impact on your life.

Activity Limitation

To begin, I would like to understand how glaucoma affects your daily activities?

*What are some of the things you could do in the past that you can’t do after having glaucoma?*

*What are some of the activities you do less because of glaucoma?*

*What other activities do you have difficulties with?*

Symptoms

First, what are some of the symptoms you experience as a result of your glaucoma?

*Can you describe your eyesight/vision?*

*How well can you see?*

*Any pain or discomfort arising from Glaucoma?*

Impact on QoL

How do your eye problems impact on your QoL?

*What areas of your life are affected by your glaucoma?*

*How much would you say your glaucoma impact on your QoL?*

*What things are harder to do because of vision loss from your glaucoma?*

*Can you think of specific examples where your glaucoma has caused you or someone else difficulty?*

*Do you need help from others for some things because of your glaucoma?*

**Depending on responses so far:**

In other groups I have talked to many people about how their glaucoma eye problems affect them emotionally. I’m interested in your experience of this.

Can you describe how your glaucoma make you feel emotionally?

*How do you feel about your eye problems?*

*Can you describe some of your emotional reactions to vision loss?*

*Do you feel like your eye condition makes you constantly worried or unhappy?*

In what ways do your glaucoma affect your social or family life?

*What things are you missing out on because of your vision?*

*What social occasions or leisure activities are affected by your vision loss?*

*How has your glaucoma affected your family and personal relationships?*

Thinking about travelling and getting out and about, can you describe how your glaucoma have affected this?

*What things are most difficult when travelling outside or in crowded places?*

*What do you find difficult when moving about in your own home?*

In what ways have your glaucoma impacted on your work life?

*What aspects of your work are harder to do?*

*Did you change or quit your job because of glaucoma?*

*How has your glaucoma impacted on you financially?*

*What things have cost you money because of your glaucoma?*

How about managing your glaucoma? In what ways does your glaucoma affect this?

*What things [e.g. reading small prints on food labels, driving at night, etc] are hard to do because of your vision loss?*

Finally, what is the ***worst thing*** about having glaucoma?

*What do you find the most annoying about your vision loss from glaucoma?*

Thank you all very much for sharing your experiences. I know this can be a difficult area to discuss but your input has been really valuable. Before we move on, I will briefly summarise what we have discussed so far………

*Is there anything else you’d like to add to this before we move on?*

Treatment and impact

I’d now like to move on to talk about eye treatments.

Has anyone had any treatment for their glaucoma? What was it?

*What were your experiences with this treatment?*

*What side effects, if any, have you experienced?*

*What is the most troublesome aspect of the treatment (eyedrops)?*

*Has the eyedrops/ laser/ surgery been effective?*

*In what ways has your treatment experience affected your QoL?*

Useful prompts to use throughout the focus group:

*Are their any other views on this?*

*Is there anything else?*

*Would you explain further?*

*Can you give me an example of what you mean?*

**Summary**

We are reaching the end of our time now. I would like to summarise the key ideas that I have heard…

*Is there anything I have missed or that you would like to add to my summary?*

*Lastly, we would like you to rank the following topics in order of importance to you/ what you are most concerned about regarding your condition. Ranking will be from 1 to 3, with 1 being the most importance/ concern and 9 being the least importance/ concern. *flash powerpoint/ hand out printed copy of diagram**

- Visual Symptoms
- Ocular Comfort
- Emotional wellbeing
- Social
- Mobility
- Economic
- Activity Limitation
- Convenience
- Health Concerns
