# Supplementary material for: The development of a glaucoma-specific health-related quality of life item bank supporting a novel computerized adaptive testing system in Asia
Source: J Patient Rep Outcomes. 2022 Oct 11;6:107. doi: 10.1186/s41687-022-00513-3 (PMC9554106; doi:10.1186/s41687-022-00513-3)
Supplement: Supplementary file 2 — Additional file 2. Supporting quotes and excerpts from focus groups showing the diverse and profound impact of glaucoma, related vision loss, and glaucoma treatments on patients’ health-related quality of life. [file 41687_2022_513_MOESM2_ESM.docx]

| **Additional File 2.** Qualitative findings about the impact of glaucoma, related vision loss, and glaucoma treatments on quality of life (n=27 participants) | | |
| --- | --- | --- |
| **Domains and example themes** | **Summary and sample quotes** | **FG/Int number, gender and language of participant** |
| ***Activity Limitation*** | *While most patients reported little difficulty reading large text or signboards, many found reading small print (e.g. letters or bills) or from a computer screen challenging. Several patients expressed reluctance to use internet banking due to lack of confidence and fear of making mistakes. Some patients also ceased engaging in sports or hobbies (e.g. playing badminton, sewing), while others reported needing more time or having to concentrate harder to complete a task.* |  |
| Reading limitations like font size, colour, or duration | “Reading a book, I easily get tired and I give up halfway of the book.”  “Same thing… nowadays I do not want to read, I just glance through that’s all… After that forget it, it doesn’t matter to me anymore because it is affecting my eyesight”  “And when there are times that I want to read, then I will try to use whatever, let’s say it is a soft copy… computer… Because when I enlarge, I can… I feel better when I read because it is not so straining on the eyes.”  “Do not know whether any of you after reading, usually I get tired in the afternoon and then I need to take a nap and then after that I can continue reading again.”  “Ya, I have that too… cannot read for too long… Maybe after 2 hours then you have to take a break because your eyes get tired.”  “So, the ‘K’, the letter ‘K’, it will no longer appear as letter ‘K’ to me.”  “It’s like a Chinese character because the center part missing.”  “For me, is when I do shopping, especially canned food, packet food, you need to read…”  “the print is let’s say light blue, and you find that you can’t see.”  “So that part is ok, it is usually reading the fine prints of the canned food or the packaging of the food because I want to know if there are any preservatives.”  “Road signs far cannot lah.” | FG1, F, Eng  FG1, M, Eng  FG, M, Eng  FG1, F, Eng  FG1, F, Eng  FG1, F, Eng  FG1, F, Eng  FG1, F, Eng  FG1, F, Eng  FG1, F, Eng  FG6, M, Eng |
| Engaging in hobbies, craft | “You want to, you know, to do some… like for myself, do some soldering on the electronic parts and all these, just for the fun of it, no more for vocation or anything.”  “You know, you just cannot slot the screwdriver into the screw head.”  “There was a day when I was playing the piano and my eyes became extremely uncomfortable.” | FG1, M, Eng  FG1, M, Eng  FG4, M, Chi |
| Playing sports | “I gave up badminton”  “Then, I cannot play football, last time I like to play football, you see. So now, all these activity is already restricted lah, I cannot do because of the eyesight, which really… year by year deteriorated I found out.” | FG5, M, Eng  FG6, M, Eng |
| Sewing | “It’s just like you cannot thread your needle.”  “Sewing. I have difficulty threading the needle” | FG1, F, Eng  FG4, F, Chi |
| Doing small, tricky tasks | “Sometimes you want to fiddle with the electrical system, you can’t see properly right?”  " Like screw, like for me ah, sometimes I repair things… small, small things I try not to do… because why, very difficult. Once the thing drop… It’s very hard to find, yes, difficult to find.” | FG1, M, Eng  FG6, M, Eng |
| Longer duration to complete task, being slower | “Because you can’t see that well, so your speed has gone down.”  ”Everything even in the housework, you have to find your things, and at my age, I think that, I also must feel that at my age, I have to go a bit slow.”  “I find that reading slower, you know.”  “The pace is slower now because I have to clearly look.”  “So, it affected my daily life. A lot of things which I suppose… I… convenient for me to do, now it’s very difficult, everything go slower.” | FG1, F, Eng  FG1, F, Eng  FG1, F, Eng  FG5, F, Eng  FG6, M, Eng |
| Filling out forms | “When you sign on very important documents, you have to read and re-read and re-read until you are very sure that it is the right document then you put your signature on it.”  “Sometimes you see all these forms, quite difficult for me also.” | FG1, M, Eng  FG2, F, Eng |
| Internet banking | “Right now I try not to go into internet things like banking you know, because I got glaucoma, the 6 can look like 8 as you say.” | FG1, F, Eng |
| Driving | “Ever since I had glaucoma, I found that driving at night was a bit harder because of the dimness of the surrounding area.”  “I think for me is more of driving got to be careful. Because especially when you are doing a right turn and could be pedestrians coming in.”  “Even if cars are coming behind you and you may want to filter, you may not see it.”  “I cannot drive at night because I cannot find words, I cannot see the addresses clearly, I miss block numbers.” | FG1, M, Eng  FG1, M, Eng  FG1, M, Eng  FG1, M, Eng |
| Grocery shopping | “Even when you go shopping or purchase something at the hotel… all the food look similar. I can hardly differentiate between pork or fish meat.” | FG3, M, Chi |
| Traveling or going on holiday | “Yes I go out less. In the past I would always travel, now much lesser. I used to travel to many countries.”  “Now, in fact I don’t… last time I like to go for holiday, you see. But now no more.” | FG3, M, Chi  FG6, M Eng |
| Picking up things/ finding things | “Yes, sometimes I struggle with picking up items.” | FG4, M, Chi |
| Recognizing family or friends | “Even sometimes when I’m working around, I cannot recognize people beyond about 5m you know.” | FG5, M, Eng |
| Unable to spend time with children | “Last time I used to help my children with their schoolwork, you know even when reading, even when drawing. I will use to help them, now totally can’t help at all, because I can’t really see.” | FG5, F, Eng |
| Unable to differentiate between colors | “Ah yeah, you cannot differentiate the colour.” | FG6, F, Eng |
| ***Visual Symptoms*** | *The most commonly reported symptom by our glaucoma patients was blurred vision, making seeing things in the distance and up close more difficult. Patients with more severe glaucoma also reported loss of peripheral vision, such as having something ‘blocking’ their vision, or experiencing a narrowing of vision.* |  |
| Blurred vision | “It’s blurred vision and even… I used to commute on buses, so with the bus… so I mistook the number and I took the wrong bus.  “whereby we start to have blurry vision. We can hardly see the person coming at us…”  “my vision is a little blurry, even though I can still identify the object”  “However, the blurry vision would appear out of nowhere, and vanish after a while.” | FG1, M, Eng  FG3, M, Chi  FG4, M, Chi  FG4, F, Chi |
| Poor vision/ double vision | “If I looked at a dim lamp, the lamp would appear to me like a flower.”  “In recent years, I started to have double vision…” | FG4, M, Chi  FG4, F, Chi |
| Floaters | “I suffered from eye floaters”  “Sometimes I feel that I can still see small dots in my eyes after surgery.” | FG4, F, Chi  FG4, F, Chi |
| Blocking of vision, tunnel vision | “It’s blank off, not there, so sometimes when I am looking for something, I cannot find it immediately.”  “So it started with my left eye, so between my left eye, 3 to 6 o’ clock, I can’t see.”  “It is a very, very narrow vision I feel.”  “Blur, not fully blur but there is always some kind of blockage like thing, where you know when you don’t see the whole thing.”  “If it is tricky I can’t even see, I have to turn my whole body to see… Like that I see nothing, both sides cannot see.”  “Yes I cannot see sideway.”  “If I try hard to focus, I can still see things. It is like walking around with a telescope.”  “What I am having trouble with is looking sideways and downward.”  “If anyone who stands at the side I may not see, I have to turn my face to see.”  “Because it’s the bad side, the one with the blocking.” | FG1, M, Eng  FG1, M, Eng  FG1, M, Eng  FG2, M, Eng  FG2, F, Eng  FG3, M, Chi  FG3, M, Chi  FG4, M, Chi  FG5, F, Eng  FG6, F, Eng |
| Smokey vision | “Because of the smoky vision that makes me cannot see, especially at night.” | FG6, M, Eng |
| ***Lighting*** | *Several patients reported that dim lighting affected daily activities like reading, walking down stairs, and driving. Bright lights were also described as too glary, with several patients reporting having to use sunglasses or a cap to block the glare. Some patients required more time adjusting their vision when moving to areas with different lighting (e.g. from areas with dark to bright lighting).* |  |
| Glare, bright lights | “I always put on sunglasses.”  “So that’s why sometimes I do put on a cap, it is to give a visor and helps to shade and helps me a lot also.”  “It is the glare that causes a lot of problems.”  “Yes, and I am afraid of light. Because of my eye, I need to wear sunglasses and this hat all the time.”  “Glare… under bright light, very glaring.”  “Also cannot, too sunny.” | FG1, F, Eng  FG1, M, Eng  FG2, M, Eng  FG3, M, Chi  FG5, M, Eng  FG6, F, Eng |
| Seeing in the dark or poor lighting conditions | “my vision is bad in evening onwards, so that is the time I need my spectacles more than day time”  “I don’t know why, but it seems even if I trip and fall, it is always in the evening onwards”  “Yes, the vision will be poorer in the night… because I tend to switch on the lights at about 4 plus in the afternoon, which is ridiculous isn’t it?"  “I have, like you, I have difficulty seeing at night.”  “I have to be very careful especially when it is dim.”  “Another thing, one problem, you go to very dark place, you go and see something very dark, you cannot see, you know. There’s total darkness, that is a fear for me.”  “Night? Sometimes you go to see the show, the whatever thing that one, they off all the light.” | FG1, F, Eng  FG1, F, Eng  FG1, F, Eng  FG1, M, Eng  FG5, F, Eng  FG5, F, Eng  FG5, F, Eng |
| Reading in dim lighting | “I find that the, I have to put it more bright otherwise I can’t read.”  “But sometimes because of lighting condition and because of the embossing of the letterings in the … the fonts in the device, cannot see so clearly, so it is very frustrating.” | FG1, F, Eng  FG1, M, Eng |
| Going down steps in dim lighting | It’s because of the specially in the nights, days not so bad, we can somehow manage but the nights, you know you miss a step, chances are you will go down falling. | FG2, M, Eng |
| Driving in different lighting conditions | “so it is only at night where the headlights, the street lights comes like a star sometimes, you know it becomes very bright, a lot of rays out.”  “It is very challenging to drive at night.” | FG2, M, Eng  FG3, F, Chi |
| Driving towards another vehicle’s headlights | “Sometimes because of the car lights shine on your face, your eyes, you somewhat a bit blur, you know.”  “The light coming from the opposite direction can be too bright and I need to cover my eyes.” | FG2, F, Eng  FG3, F, Eng |
| Seeing in indoor lighting conditions | “Indoor lighting is less of a concern, as it is less intrusive.” | FG3, M, Chi |
| Adjusting vision from a dark to right area or vice versa | “I used to wear brown sunglasses indoors, because it transitions very well from outdoors to indoors.” | FG4, F, Chi |
| ***Mobility& Independence*** | *A decrease in peripheral vision posed challenges to patients as they moved around their community. Walking on uneven ground, navigating crowded areas, and seeing people or objects coming towards them were particularly problematic.* |  |
| Seeing objects coming towards you | “But, the trouble with this glaucoma is this… especially when we are not that alert, an e-bike comes past by you, you can’t see that you know, suddenly you can’t see that.”  “I have many times being run into by e-scooters.” | FG1, M, Eng  FG4, M, Chi |
| Noticing things to the left or right when walking, | “It is a very, very narrow vision I feel.”  “I think there is on one incident when I was walking along Chinatown, and on my left, I can’t… I mean, sorry on my right side, I can’t see people coming by, and they always bang into me.”  “I bump into tables when I walk around a room”  “I noticed this, is because sometimes somebody is standing, I will just move very fast and I hit very hard, you know. Without knowing that the person is standing beside me. | FG1, M, Eng  FG1, F, Eng  FG4, F, Chi  FG5, F, Eng |
| Walking down stairs or steps | “I tend to trip and fall, so what I tend to do now when I walk, I don’t look up, I just look down so that I know there’s steps, there are kerbs.  “Whenever I walk down the staircase especially, walking up is no problem.”  “Walking down the staircase, I need to hold onto the handrails… Otherwise I may miss a step and just tumble.”  “You will struggle to see the steps in staircases.”  “I have fallen three time before, simply because I failed to see what is underneath.” | FG1, F, Eng  FG1, M, Eng  FG1, M, Eng  FG3, M, Chi  FG4, M, Chi |
| Using unmarked steps | “I think one issue sometimes I face is steps or when there is a little drop and the steps have got the same color… When you are from the top, you don’t realize there is a drop and so you take it as the same level then suddenly you get injured, you know?”  “You saw all the same colors, you not sure whether there is a step there or no step.”  “But if everything is the same, that’s why I observe certain places they have yellow lines, maybe that is an indication to say that there is something there, you got to be careful”  “On the ground sometimes you will see yellow line. This is a colored marker to notify us.”  “I found out ah, government would do yellow line, you know. Then I can differentiate… that is a kerb.” | FG1, M, Eng  FG2, M, Eng  FG2, M, Eng  FG3, M, Chi  FG6, M, Eng |
| Walking on uneven ground | “There is a lot of bumps, so you miss and you start missing your step, that is the problem.”  “Then at night when I walk, sometimes when… if the path is not level, I’m not able to see the gap.”  “But only, I go out I’m scared because levelling I cannot see properly.” | FG2, M, Eng  FG5, M, Eng  FG6, F, Eng |
| Walking outdoors | “If I go outdoor, then it can be quite troublesome.”  **“**So walking outdoors is a challenge and I could not cross the road myself.”  **“**I have issue walking. I am constantly worried.”  **“**I have to be more careful, but if I don’t have to go out, I don’t go” | FG3, F, Chi  FG4, F, Chi  FG4, M, Chi  FG5, F, Eng |
| Crossing the road | “Even when I am crossing the road, I have to be very careful.”  “Crossing the road, yes, for me lah… I always have to be very careful. I always find a way where there’s a zebra crossing, or maybe I have to stay and look very clearly. I mean, I have to go slow lah. Be alert lah most of the time.”  “Same, save me for crossing also lah because you see only here, but you cannot see side.” | FG5, F, Eng  FG6, M, Eng  FG6, F, Eng |
| Getting on or off a vehicle | “Because one time, I would take taxi, then I… the step is down I didn’t tell, almost I drop down already what, lucky got people help me because I levelling is very, very bad.” | FG6, F, Eng |
| Going to an unfamiliar place | “Maybe the new place you go, maybe you find a bit difficult. Difficult and I think, scared also.” | FG6, F, Eng |
| ***Psychosocial*** | *A universal fear reported by glaucoma patients was further loss of vision and eventual blindness. Most patients expressed safety concerns, like falling, tripping, or bumping into people or objects. Many also shared worries of losing independence and reluctance about having to rely on family for help. Similarly, many patients often felt frustrated and drained with their glaucoma, with some patients confiding that there were moments where they wanted to ‘give up’ on their eye condition and treatment.* |  |
| Helpless, a sense of loss | “You know, the quality of life is gone… Yes, because every now and then I have to lie down on my bed… What’s the use?”  “It’s so hard to work, so what can I do?”  “There is nothing much I can do.”  **“**Glaucoma is an invisible killer.” | FG1, M, Eng  FG1, M, Eng  FG1, M, Eng  FG4, F, Chi |
| Family or people not understanding | “And some men are so aggressive and then I said I am sorry, I got bad eyesight I told him, and then he just move on.  “So this is quite an irritation to my wife.”  “Easier for me freedom for me to go alone, with someone if they are not patient.”  “I almost collide into someone. That person was frustrated at me, blaming me for the collision.” | FG1, F, Eng  FG1, M, Eng  FG2, F, Eng  FG3, M Chi |
| Fear of falling, tripping, bumping | “I’m a little younger than you guys, but I actually had this fear of falling down when I go down the steps which I didn’t have before.” “I used to be able to hop down, run up, you know I still run up, but nowadays I’m a little careful, like what you say, I would hold on to the railings, because I suddenly had this hunch that I might tumble down.”  “For me, it doesn’t really affect my quality of life but there is a slight concern because of this glaucoma, the concern is I may slip and fall when I miss my step when walking down steps you know.”  “Nowadays I do not even dare to step outside. You know why? We are worried of falling down.”  “For me, in my daily life, I trip quite a lot because of my poor vision.” | FG1, F, Eng  FG1, F, Eng  FG1, M, Eng  FG3, M, Chi  FG5, M, Eng |
| Fear of becoming blind | “Blindness is for sure… That is the main concern.”  “You come to a point when I will be blind in one eye.”  “We are just worried that we would go blind, and like what she said, suffer from occasional lost of eyesight.”  “Fear of losing my eyesight.”  “Yeah! Scared become blind, what.” | FG1, M, Eng  FG1, M, Eng  FG4, F, Chi  FG5, F, Eng  FG6, F, Eng |
| Failing vision, or eyesight getting worse | “In fact I worry a lot about my vision because I find it, you know very difficult to do things”  “You always worry about the next test that you come, whether your eyes deteriorate because you can’t tell.”  “My one side is very bad already. Now it’s going worse, I did implants already, I think last October replant me the tube also. Doesn’t help much. It’s becoming worse already.” | FG2, F, Eng  FG2, M, Eng  FG6, F, Eng |
| Loss of confidence in usual activities | “If you do shopping, worse… I tell you, you go supermart, you go blind.” | FG2, F, Eng |
| Frustration, annoyance | “Very frustrating.”  “Ya, these are some of the things, we so used to it, you know, and find that eh suddenly these are taken away…Frustrated.”  “My greatest frustration was when I first heard I got glaucoma, so I asked the doctor if there is anything I can do to at least slow down the progression, and the answer is ‘No.’” | FG1, M, Eng  FG1, M, Eng  FG1 F, Eng |
| Angry | “I feel very frustrated that I got to go here go there, you know, doing all these check-ups, so then I got fed up with you know why my eyes like that.”  “Oh, if you really cannot see… you yourself get angry. Yes, I get angry! I’m very angry. Myself.” | FG2, F, Eng  FG6, F, Eng |
| Sad | “Sometimes I still cannot get over the fact that I suffer from glaucoma.”  “I find it very sad and horrifying.” | FG4, F, Chi  FG4, F, Chi |
| Depressed | “You can see and then after that you go blind…  You will feel depressed. So that time during that time I feel like depressed lah.” | FG6, M, Eng |
| Feeling unfairness | “Definitely. I often wonder why, among all my siblings, it was me who suffer from glaucoma?” | FG4, F, Cho |
| Afraid of not being able to certain activities in the future | “Imagine not being able to do anything. As a booklover, I will not be able to read any book.” | FG4, F, Chi |
| Emotionally Drained | “It’s more mental than anything, physically it does not affect me at all.” | FG2, M, Eng |
| Regretful about eyecare in the past | “I believe when I think back how I got glaucoma, is lifestyle. Because in the early days, I used to sleep late, happen to do my things and all that.”  “So probably like what you say, the eye strain, the long hours on the computer that could have affected it.”  “This is because we abuse our eyes.” | FG1, M, Eng  FG1, M, Eng  FG3, M, Chi |
| Worried about the future | “I mean like basically is whether is it going to get worse because I cannot envision myself going through life not being able to see.” | FG2, M, Eng |
| Feels like glaucoma is affecting many areas of life | “I like to watch TV, I would like to see my children, my grandchildren, things like that. I like to travel you know, and if you can’t see, then the quality of life is not there anymore, you know.” | FG2, M, Eng |
| Limitation of social activities | “For me, I try to get home before night because as I said, night time, my vision is bad, so that is the… I try to play safe, not going out too late… So this is maybe one of the social functions that I have to cut down.”  “I go out less nowadays. That is my current situation.”  “And then, my social activity, I don’t socialise more like last time.” | FG1, F, Eng  FG3, M, Chi  FG6, M, Eng |
| Losing driver’s license | “That’s why I gave up my license.”  “Yes, the doctor told me not to drive, so I listened. I handover my license to LTA and asked them to terminate it.  “I don’t drive due to this double vision. Initially I could, now I can no longer do so.” | FG2, F, Eng  FG4, M, Chi  FG4, F, Chi |
| Losing independence in the future or having to rely on others for help | “I’m afraid that in the future, should my 2 daughters not with me, then you know, there will be a big problem.”  “Going outside I need people’s help… but some new places I must bring somebody along, my sister or whatever”  “I have to take help from my husband, my children everybody have to accompany me you know.” | FG1, F, Eng  FG6, F, Eng  FG6, F, Eng |
| Missing out on things in life/ social isolation | “In the past I love to go out with friends, anywhere they go, I will follow.”  “Traveling but now no more, 20 years ago no more traveling.” | FG2, F, Eng  FG2, F, Eng |
| Being a burden to family | “How can I expect him to bring me to the hospital? He still has to work right?” | FG3, M, Chi |
| Concerns about passing glaucoma to your children | “My greatest worry is because it is hereditary, so I’m worried that I know my kids will get it.” | FG1, F, Eng |
| Concerns about side effects and effectiveness of treatment | “We want to know whether the eye drops will affect… give us any other problems.”  “The thing is that sometimes actually we don’t really know whether it is getting better, I don’t think it is getting better, just hope that it is what we are right now.”  “Is it a stable kind of treatment?”  “You cannot ask the doctor, how is my eye, is it get better?” | FG1, M, Eng  FG2, F, Eng  FG2, M, Eng  FG5, F, Eng |
| Cost of treatment and appointments | “Eye drops are really expensive.”  “If you project, actually we are going to spend quite a fair bit.”  “even though these are subsidized rates but it is still a fair bit.”  “And that bottle is not a cheap…”  “I think the eye drops rather expensive.”  “I think the consultation fee is a lot you know.”  “So the eye drop was crazily expensive. Around 50 dollar per bottle.”  “The medication can be very expensive, and the consultation cost adds up too.” | FG1, F, Eng  FG1, M, Eng  FG1, M, Eng  FG1, F, Eng  Fg2, F, Eng  FG2, F, Eng  FG3, M, Chi  FG4, F, Chi |
| On having to undergo surgery | “I can see already, I go back to the doctor and say, “I don't want to operate because I can see clearly, why must I to operate?” | FG4, F, Chi |
| ***Ocular Comfort*** | *People reported feeling like there was something in their eyes or having dry eyes. Some patients reported experiencing episodes of headaches or pain around their eyes, or red eyes. The prolonged use of eyedrops also caused side effects, such as a sticky sensation around their eyelashes or eyelids, or longer and thicker eyelashes.* |  |
| Eye symptoms | “It becomes red.”  “Sometimes can be painful.”  “Because I know that for Xalatan when it is effective, it stings when you first apply”  “I have itch around my eyelid, you know.”  “I find my eyes are very dry.”  “Actually I have dry eyes and then the… I got pain in the eyes, like you know, the needle poking your eyes like that.”  “One day one of my eyes was in pain.”  “People would look at me and ask why I have red eyes.”  “they turned red again after a month and did not recover at all after one week.”  “I will feel pain and dry.”  “It just turned red, you know.”  “Itchy eyes… yes!” | FG1, M, Eng  FG1, F, Eng  FG1, F, Eng  FG1, M, Eng  FG2, F, Eng  FG2, F, Eng  FG3, M, Chi  FG3, F, Chi  FG4, F, Chi  FG4, F, Chi  FG4, F, Chi  FG6, F, Eng |
| Stickiness in eyelashes or eyelids | “Ya, ya… after 45 min or an hour, you find that your eyes seem to be glued all, you know. Your eyelids just cannot move you know.”  “Tightness.”  “can’t open the eyes, simply cannot, you have to force it out a bit.” | FG1, M, Eng  FG1, F, Eng  FG1, M, Eng |
| Change in eye appearance or eyelashes | “The eye bags will turn dark and the eyelashes will grow longer.”  “Yes, the eyelashes really grow longer.” | FG4, M, Chi  FG4, F, Chi |
| Headaches | “and I had severe headache out of the blue.”  “Headache, I do have.” | FG3, M, Chi  FG5, F, Eng |
| Tired eyes | “I can still perform most of the activities, just that my eyes would become very tired”  “My eyes would feel very dry and very tired.” | FG3, M, Chi  FG3, M, Chi |
| Allergic reaction to eye drops | “Yes I have changed my eye drop. After the switch, my eyes became swollen and I had to rush to the hospital. My eyes become very red and tears keep flowing out of my eyes.”  “Some of the eye drops… I am allergic to one of the eye drops.” | FG3, F, Chi  FG4, F, Chi |
| Stinging in eyes | “The effect is stinging.”  “Well, after putting the eye drops, yeah little pain, that is common I think after putting drops, little pain is there.” | FG5, M, Eng  FG6, F, Eng |
| Feeling like there is something in eye | “I feel like there’s a sand or what like that… irritating ah..” | FG6, F, Eng |
| ***Glaucoma Management*** | *The administration of eyedrops multiple times a day proved to be tiresome for some patients, and several reported that they had difficulty remembering to take their eye drops. Some patients wondered whether their treatment plan was effective, while others were concerned about having to undergo surgery or laser treatment for their glaucoma and the associated financial burden.* |  |
| Taking your glaucoma medication when travelling or out of the house | “We have to carry it with us wherever we go. That is one of the setbacks.”  “Every night, even when I travel in the plane I also bring along with the ice pack.”  “In case a day comes when I go overseas, I travel and I forget to bring my eye drops… And if I am in another country, I can’t just go to a drugstore and buy the eye drops, they will never give it to me.”  “Initially when I travel I used to carry ice packs and look for hotel with refrigerator because … or without it, you have to go to the what you call it…down to the cooking area where they have refrigerators.”  “sometimes when you are outdoor it is very inconvenient to apply eye drop.”  “Sometimes you were outside, or had dinner appointments, which were not the most convenient timing to apply eye drop.”  “I have to carry my eye drops wherever I go… Inconvenience, it has to be with me all the time.” | FG1, M, Eng  FG1, F, Eng  FG1, M, Eng  FG2, M, Eng  FG3, M, Chi  FG4, F, Chi  FG5, F, Eng |
| Difficulty administering eye drops, or the right amount of drops | “But today I put the drops, the thing flow down my cheeks then I know that I realized that, “oh, I got the drop already. That’s why I tend to use more.”  “But sometimes if you drop sideways, you still have to do it again right?”  “It is best that you put your eye drops when you are lying down on the bed, ok.”  “Is there a better way of putting eye drops because sometimes I put in, then you blink, then obviously it comes out.” | FG1, F, Eng  FG1, F, Eng  FG1, M, Eng  FG5, M, Eng |
| Remembering to administer eye drops, fitting drops into daily routine | “Sometimes I skip you know... Lazy…I just want to sleep.”  “And then on top of that, last time I used to miss out on my eye drops, so now I do a chart.”  “That part is also quite worrying if you forget to put the eye drop.”  “Trusopt I am supposed to use twice a day, night and morning but I only use night because every morning I forget.”  “I try to be every day but sometimes I may skip 1, 2 days because I forget. | FG1, M, Eng  FG1, M, Eng  FG1, F, Eng  FG2, M, Eng  FG2, F, Eng |
| Using several different eye drops | “For the full month, because for my case, I have 2 drops of Timolol and plus 1 drop of … The last time I used to forget, I used to duplicate.”  “My situation is a little different. I have one eye drop specific for my right eye, and another one specific for my left eye. Very troublesome.” | FG1, M, Eng  FG3, M, Chi |
| Applying eye drops several times a day | “Right now I am relying on this eye drop. The challenge I have is with the frequency, as I need to apply it every two hours.”  “I have been using the 3 for a couple of years. Make sure I got to put it at the right time. So timing is very important, so I must make sure I put in.” | FG4, F, Chi  FG5, F, Eng |
| Discharge around eyes | “Particularly the two corners. Some whitish thing would come out and we could squeeze more out as well.”  “There are whitish secretion.” | FG4, F, Chi  FG4, F, Chi |
| Performing routine eye tests at each glaucoma appointment | “It is difficult for me, because my right eye, the sense of vision is gray so I don’t see the orange light.”  “Meanwhile for eye pressure, I need to come to the hospital. It is very troublesome. I cannot measure my own eye pressure.” | FG2, M, Eng  FG4, F, Chi |
| Change in treatment regime | “But because the problem is sometimes when your eyes deteriorate, either your medicines are going to be changed, or if not some other things happen and you are stuck with the rest, you can’t return the medicine.”  “But if you say side effects, I put this medicine, I changed so many medicine, you know.” | FG2, M Eng  FG5, F, Eng |
| Seeing different eye doctors at each appointment/ receiving different opinions from different eye doctors | “So I don’t really have the… you know, the ability to talk to her or to find out exactly what is happening. Cos I am seeing different doctors every time. And different doctors have different views and think… they look at it differently.”  “The doctors keep on changing, so I don’t know whether you are actually getting the right treatment as such. So you are a bit concerned that your case is not being followed up by the same person who understands your condition.”  “I think I also would prefer the same doctor.” | FG2, M, Eng  FG2, M, Eng  FG2, F, Eng |
| Amount of time needed to attend eye appointments | “The consultation time is generally very long, and now it has been extended into two days. Now all consultations need at least two days. In the past it can be completed within the same day.” | FG3, F, Chi |
| ***Work*** | *Difficulties reading and working on a computer screen for long hours impacted the work performance of some glaucoma patients. Several patients had to switch jobs or retire early due to their failing eyesight. Of those still in the workforce, a few reported keeping their eye condition a secret from their employers for the fear of losing their jobs. Work relationships with colleagues or bosses were also strained due to their inability to perform certain work tasks or having to take leave for their eye appointments* |  |
| Difficulties with work tasks | “But it does affect me because I am looking after building projects, so we do look at building drawings and you know drawings there are words that can be very small, lines you know.”  “It is just sometimes the presentation on the wall may not be clear, because the fonts may be a little small, so that is a bit straining.”  “Frustration is when I am working. When the eyes start tearing, I can’t read.”  “I have to be very careful because I have to make sure patient’s particulars, documents that I see must be correct, so I have to be extra careful.”  “I think impacted quite a lot because I need to move my computer screen nearer… So I need to enlarge the fonts, sometimes I have to go near and look at the figures.” | FG1, M, Eng  FG1, M, Eng  FG5, F, Eng  FG5, F, Eng  FG5, M, Eng |
| Not being able to do certain work, financially affected by job loss | “I am a Uber driver, I am private hire, so because I could not see at night, I could find the addresses.”  “So it is a huge loss of income for me.”  “In fact, last time I can work. So specifically, I can work but now I cannot work.” | FG1, M, Eng  FG1, M, Eng  FG6, M, Eng |
| Abbreviations: Chi=Chinese-speaking; Eng=English-speaking; F=female; FG=focus group; M=male | | |
