# Supplementary material for: The development of a glaucoma-specific health-related quality of life item bank supporting a novel computerized adaptive testing system in Asia
Source: J Patient Rep Outcomes. 2022 Oct 11;6:107. doi: 10.1186/s41687-022-00513-3 (PMC9554106; doi:10.1186/s41687-022-00513-3)
Supplement: Supplementary file 3 — Additional file 3. Sociodemographic and clinical characteristics of the 27 participants who participated in the cognitive interviews to pre-test the GlauCAT™-Asian instrument. Most patients were male, Chinese and had received topical medication in at least one eye. [file 41687_2022_513_MOESM3_ESM.docx]

| **Additional File 3.** Sociodemographic and clinical characteristics of the 27 participants in Phase 2 | | |
| --- | --- | --- |
| **Variable** | **N** | % |
| *Gender* |  |  |
| Male | 16 | 59.3% |
| Age (Years) |  |  |
| 40 - 49 | 3 | 11.1% |
| 50 - 59 | 3 | 11.1% |
| 60 - 69 | 11 | 40.7% |
| 69 < | 10 | 37.0% |
| *Ethnicity* |  |  |
| Chinese | 24 | 88.9% |
| Malay | 1 | 3.7% |
| Indian | 2 | 7.4% |
| Duration of glaucoma |  |  |
| 0-2 | 4 | 14.8% |
| 3-5 | 9 | 33.3% |
| 6-10 | 6 | 22.2% |
| 11-15 | 6 | 22.2% |
| >15 | 1 | 3.7% |
| Allergic reactions/side effects to medication |  |  |
| Yes | 4 | 14.8% |
| No | 23 | 85.2% |
| *Glaucoma type (per eye)* |  |  |
| POAG/ NTG | 34 | 63.0% |
| PACG | 12 | 22.2% |
| None | 8 | 14.8% |
| *Glaucoma Severity (better eye)* |  |  |
| None | 9 | 33.3% |
| Mild | 6 | 22.2% |
| Moderate | 6 | 22.2% |
| Severe | 4 | 14.8% |
| Advanced/End-stage | 2 | 7.4% |
| *Glaucoma Severity (worse eye)* |  |  |
| Mild | 6 | 22.2% |
| Moderate | 6 | 22.2% |
| Severe | 4 | 14.8% |
| Advanced/End-stage | 11 | 40.7% |
| *Glaucoma treatments (in at least one eye)* |  |  |
| Topical medication | 21 | 77.8% |
| Laser | 7 | 25.9% |
| Surgery | 8 | 29.6% |
| *Vision impairment (better eye)* |  |  |
| None (≤0.3 LogMAR or ≤20/40 Snellen) | 27 | 100.0% |
| Mild (>0.3 LogMAR ≤0.48 or >20/40 Snellen ≤20/60 ) | 0 | 0.0% |
| Moderate/severe (>0.48 LogMAR or >20/60 Snellen) | 0 | 0.0% |
| *Vision impairment (worse eye)* |  |  |
| None (≤0.3 LogMAR or ≤20/40 Snellen) | 16 | 59.3% |
| Mild (>0.3 LogMAR ≤0.48 or >20/40 Snellen ≤20/60 ) | 3 | 11.1% |
| Moderate/severe (>0.48 LogMAR or >20/60 Snellen) | 8 | 29.6% |
| *Marital status* |  |  |
| Single | 8 | 29.6% |
| Married | 16 | 59.3% |
| Divorced/separated/widowed | 3 | 11.1% |
| *Highest Education level^a^* |  |  |
| Primary | 0 | 0% |
| Secondary | 9 | 33.3% |
| A Level | 3 | 11.1% |
| Polytechnic/Diploma/ Vocational Training | 9 | 33.3% |
| University or higher | 6 | 22.2% |
| *Employment status* |  |  |
| Working | 15 | 55.6% |
| Not working | 12 | 44.4% |
| Chronic health conditions^a^ |  |  |
| Hypertension | 11 | 40.7 % |
| Dyslipidaemia | 9 | 33.3% |
| Diabetes | 3 | 11.1% |
| Heart attack | 0 | 0.0% |
| Stroke | 0 | 0.0% |
| **Continuous variables** | **Mean** | **SD** |
| Age (years) | 65.8 | 11.3 |
| Presenting VA (better eye), LogMAR; Snellen | 0.11; 20/25 | 0.10; 20/25 |
| Presenting VA (worse eye), LogMAR; Snellen | 0.31; 20/40 | 0.23; 20/32 |
| Visual fields (better eye), mean deviation | -7.51 | 6.84 |
| Visual fields (worse eye), mean deviation | -15.51 | 10.11 |
| No. of topical treatments within past 6 months | 1.2 | 0.9 |
| ^a^Percentages for some variables may not equal 100% due to missing data or participants selecting >1 category  LogMAR=Logarithm of the minimal angle of resolution; NTG=Normal tension glaucoma; PACG=Primary angle closure glaucoma; POAG=Primary open angle glaucoma; SD=Standard deviation; SGD=Singapore dollars; VA=visual acuity | | |
