# Supplementary material for: The development of a glaucoma-specific health-related quality of life item bank supporting a novel computerized adaptive testing system in Asia
Source: J Patient Rep Outcomes. 2022 Oct 11;6:107. doi: 10.1186/s41687-022-00513-3 (PMC9554106; doi:10.1186/s41687-022-00513-3)
Supplement: Supplementary file 4 — Additional file 4. Comparison of the content (domains and items) covered in the GlauCAT™-Asian instrument and related glaucoma-specific quality of life questionnaires and item banks, showing that some domains (e.g. Activity Limitation, Lighting, Mobility, and Psychosocial) were reasonably well represented in related questionnaires, while others (e.g. Ocular Comfort Symptoms, Glaucoma Management and Work) were less well covered. [file 41687_2022_513_MOESM4_ESM.docx]

| **Additional File 4.** Content coverage of our GlauCAT^TM^-Asian instrument compared to related glaucoma-specific quality of life questionnaires and item bank* | | | | | | | | | | | |
| --- | --- | --- | --- | --- | --- | --- | --- | --- | --- | --- | --- |
| **Domain#** | **GlauCAT^TM^-Asian** | **Matsuura et al item bank^1^** | **GQL-15^2^** | **Glau-QoL^3^** | **AGQ^4^** | **Concerns Q^5^** | **IMQ^6^** | **TSS-IOP^7^** | **COMTOL^8^** | **GSI^9^** | **GSS^10^** |
| Ocular comfort symptoms | Yes (19) | Yes (2) | No | No | No | No | No | Yes (4) | Yes (5) | No | Yes (6) |
| Activity Limitation (including driving) | Yes (72) | Yes (84) | Yes (4) | Yes (10) | Yes (9) | No | No | No | Yes (5) | Yes (17) | No |
| Lighting | Yes (15) | Yes (8) | Yes (5) | No | Yes (5) | No | No | No | No | Yes (6) | Yes (3) |
| Mobility & Independence | Yes (19) | Yes (15) | Yes (6) | No | Yes (8) | No | Yes (35) | No | No | Yes (9) | No |
| Psychosocial | Yes (55) | Yes (17) | No | Yes (15) | Yes (9) | Yes (10) | No | Yes (3) | No | No | No |
| Glaucoma Management | Yes (28) | Yes (5) | No | Yes (11) | No | No | No | Yes (8) | No | No | No |
| Work | Yes (13) | Yes (5) | No | No | No | No | No | No | No | No | No |
| *Selected on the basis of strong psychometric properties; #The item content of each instrument was directly matched to the GlauCAT^TM^-Asian domains and does not necessarily reflect the domain structure reported by the instrument developers.  AGQ=Aberdeen Glaucoma Questionnaire; COMTOL=Comparison of Ophthalmic Medications for Tolerability; Glau-QoL=Glaucoma Quality of Life; GQL-15=Glaucoma Quality of Life-15 items; GSI=Glaucoma Symptom Identifier; GSS=Glaucoma Symptom Scale; IMQ=Independent Mobility Questionnaire; TSS-IOP=Treatment Satisfaction Survey for Intra Ocular Pressure; | | | | | | | | | | | |

**References**

1. Matsuura M, Hirasawa K, Hirasawa H, Yanagisawa M, Murata H, Mayama C, Asaoka R. Developing an Item Bank to Measure Quality of Life in Individuals With Glaucoma, and the Results of the Interview With Patients: The Effect of Visual Function, Visual Field Progression Rate, Medical, and Surgical Treatments on Quality of Life. *Journal of glaucoma* 2017; **26**(2): e64-e73.

2. Nelson P, Aspinall P, O'Brien C. Patients' perception of visual impairment in glaucoma: a pilot study. *Br J Ophthalmol* 1999; **83**: 546-52.

3. Bechetoille A, Arnould B, Bron A, Baudouin C, Renard JP, Sellem E, Brouquet Y, Denis P, Nordmann JP, Rigeade MC, Bassols A, Benmedjahed K, Guillemin I, Rouland JF. Measurement of health-related quality of life with glaucoma: validation of the Glau-QoL 36-item questionnaire. *Acta Ophthalmol Scand* 2008; **86**(1): 71-80.

4. Prior M, Ramsay CR, Burr JM, Campbell SE, Jenkinson DJ, Asoaka R, Francis JJ. Theoretical and empirical dimensions of the Aberdeen Glaucoma Questionnaire: a cross sectional survey and principal component analysis. *BMC ophthalmology* 2013; **13**: 72.

5. Mogil RS, Tirsi A, Lee JM, Tello C, Park SC. Glaucoma Patient-Reported Concerns and Associated Factors. *American journal of ophthalmology* 2017; **178**: 9-17.

6. Turano KA, Massof RW, Quigley HA. A self-assessment instrument designed for measuring independent mobility in RP patients: generalizability to glaucoma patients. *Investigative ophthalmology & visual science* 2002; **43**(9): 2874-81.

7. Atkinson MJ, Stewart WC, Fain JM, Stewart JA, Dhawan R, Mozaffari E, Lohs J. A new measure of patient satisfaction with ocular hypotensive medications: the Treatment Satisfaction Survey for Intraocular Pressure (TSS-IOP). *Health Qual Life Outcomes* 2003; **1**: 67.

8. Barber BL, Strahlman ER, Laibovitz R, Guess HA, Reines SA. Validation of a questionnaire for comparing the tolerability of ophthalmic medications. *Ophthalmology* 1997; **104**(2): 334-42.

9. Walt JG, Rendas-Baum R, Kosinski M, Patel V. Psychometric evaluation of the Glaucoma Symptom Identifier. *Journal of glaucoma* 2011; **20**(3): 148-59.

10. Lee BL, Gutierrez P, Gordon M, Wilson MR, Cioffi GA, Ritch R, Sherwood M, Mangione CM. The Glaucoma Symptom Scale. A brief index of glaucoma-specific symptoms. *Arch Ophthalmol* 1998; **116**(7): 861-6.
